# Left ventricular pacing vector selection by novel echo-particle imaging velocimetry analysis for optimization of quadripolar cardiac resynchronization device: a case report

**DOI:** 10.1186/s13256-016-0965-9

**Published:** 2016-07-01

**Authors:** Alfonso/A. Roberto/R. Martiniello, Gianni/G. Pedrizzetti, Valter/V. Bianchi, Giovanni/G. Tonti, Antonio/A. D’Onofrio, Pio/P. Caso

**Affiliations:** 1 Department of Cardiology, Monaldi Hospital, AORN Ospedali dei Colli, Via Leonardo Bianchi, 80131 Naples, Italy; 2 Department of Engineering and Architecture, University of Trieste, Trieste, Italy; 3 Institute of Cardiology and Center of Excellence on Aging, ‘G. d’Annunzio’ University , Chieti, Italyᅟ

**Keywords:** CRT, Quadripolar lead, Echo-PIV, Fluid dynamics, Vortex, RT3D-TTE, Case report

## Abstract

**Background:**

The availability of pacing configurations offered by quadripolar left ventricular leads could improve patients’ response to cardiac resynchronization therapy; however, the selection of an optimal setting remains a challenge. Echo-particle imaging velocimetry has shown that regional anomalies of synchrony/synergy of the left ventricle are related to the alteration, reduction, or suppression of the physiological intracavitary pressure gradients. These observations are also supported by several numerical models of the left ventricle that have shown the close relationship between wall motion abnormalities, change of intraventricular flow dynamics, and abnormal distribution of forces operating on the ventricular endocardium.

**Case presentation:**

A 73-year-old white man in New York Heart Association III functional class with an ejection fraction of 27.5 % did not improve after 1 month of cardiac resynchronization therapy. Five configurations were tested and settings were defined by optimizing intraventricular flow. After 6 months, he became New York Heart Association II class with left ventricular ejection fraction of 53.2 %.

**Conclusions:**

The abnormal dynamic of pressure gradients during the cardiac cycle, through biohumoral endocrine, autocrine, and paracrine transduction, may lead to structural changes of the myocardial walls with subsequent left ventricular remodeling. The echo-particle imaging velocimetry technique may be useful for elucidating the favorable effects of cardiac resynchronization therapy on intraventricular fluid dynamics and it could be used to identify appropriate pacing setting during acute echocardiographic optimization of left pacing vector.

**Electronic supplementary material:**

The online version of this article (doi:10.1186/s13256-016-0965-9) contains supplementary material, which is available to authorized users.

## Background

Unfavorable response to cardiac resynchronization therapy (CRT) is determined by different causes, such as an inadequate selection of patient, or issues related to the implant of the device such as a non-optimal position of the lead in the coronary sinus or an incorrect programming of biventricular pacemaker.

The availability of pacing configurations offered by quadripolar left ventricle (LV) leads could improve a patients’ response; however, selection of an optimal setting remains a challenge. Recent studies suggested that images of LV flow by echo-particle imaging velocimetry (echo-PIV) could be a useful marker of synchrony [1], which could be considered for pacing optimization [2]. This is the first report in the literature that emphasizes the value of intraventricular flow analysis in CRT optimization. In this remarkable case report, flow analysis allowed us to get a response from an otherwise unsuccessful therapy by analyzing changes in size and direction of two-dimensional intraventricular pressure gradients induced by CRT with a quadripolar LV lead.

## Case presentation

**Fig. 1:**
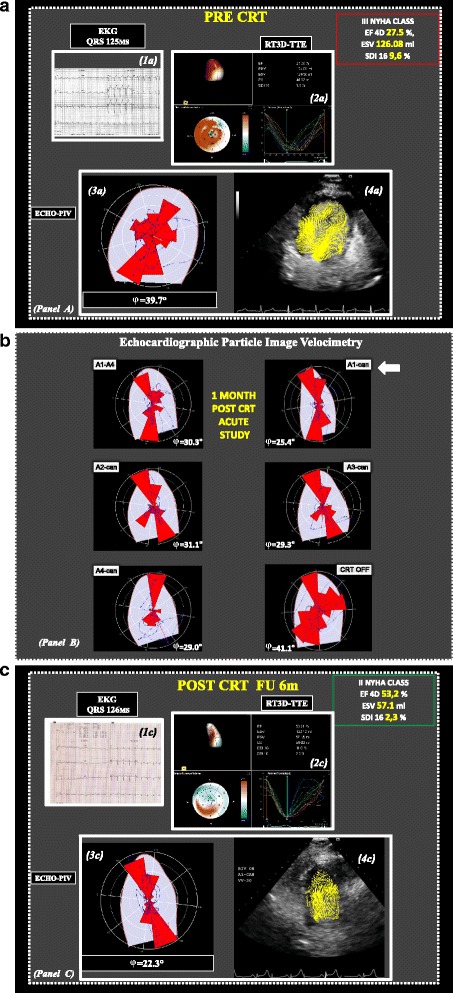
Panel **a** *1a* Twelve-lead electrocardiograms of a patient with heart failure pre-cardiac resynchronization therapy demonstrates a QRS duration of 125 ms. *2a* Real-time three-dimensional transthoracic echocardiography full volume mode, one beat: very heterogeneous (*orange regional pattern*) dynamic map of the time minimum volume that looks at a 10 % of heart cycle time window when it moves through the heart cycle, with individual segments reaching end-systole at different times, and relative high Systolic Dyssynchrony Index of 9.6 %. *3a* The polar histogram shows the orientation and relative magnitude of blood-induced intraventricular forces which are not properly aligned along the left ventricle axis. *4a* Relative magnitude of blood-induced hemodynamic forces which are not properly aligned along the left ventricle axis. Panel **b** Changes in electrical activation settings modify the orientation, φ, of intraventricular forces during acute study. The setting (*A1-can in the top right, arrow*) corresponding to the most aligned intraventricular forces is selected. Panel **c** *1c* At follow-up, 12-lead electrocardiograms post-cardiac resynchronization therapy demonstrate a QRS duration of 126 ms. *2c* Real-time three-dimensional transthoracic echocardiography full volume mode, one beat: low heterogeneous (*orange regional pattern*) dynamic map of the time minimum volume that looks at a 10 % of heart cycle time window when it moves through the heart cycle, with individual segments reaching end-systole at similar times, with low Systolic Dyssynchrony Index of 2.3 %. *3c* These settings provided a positive response to therapy, which was associated with improved alignment of intraventricular forces. *4c* Left ventricle flow was more regular and the associated hemodynamic forces followed the base–apex orientation. *CRT* cardiac resynchronization therapy, *echo-PIV* echo-particle imaging velocimetry, *EF* ejection fraction, *EKG* electrocardiogram, *ESV* end-systolic volume, *NYHA* New York Heart Association, *RT3D-TTE* real-time three-dimensional transthoracic echocardiography, *SDI* Systolic Dyssynchrony Index

A 73-year-old white man with non-ischemic dilated cardiomyopathy diagnosed for at least 2 years, in functional New York Heart Association (NYHA) class III despite optimal medical therapy, with left bundle branch block and a QRS duration of 125 msec (Fig. 1: 1a) was evaluated for CRT with defibrillator (CRT-D) device implant. A pre-implant transthoracic echocardiographic examination was performed with infusion of contrast agent to improve diagnostic accuracy in the assessment of LV systolic function and wall motion analysis. The real-time three-dimensional transthoracic echocardiographic (RT3D-TTE) approach using the “full-volume” mode showed an ejection fraction (EF) of 27.5 %, an end-systolic volume (ESV) of 126 ml, and a high Systolic Dyssynchrony Index (SDI) 9.6 % with a more delayed mechanical activation in the basal-middle-apical posterior and anterior interventricular septum segments of his LV, respectively (Fig. 1: 2a; see Additional file 1 for video).

We implanted a CRT-D device with a quadripolar LV lead placed in the posterolateral branch of his coronary sinus. After recording the right ventricle (RV)-to-LV electrical delay [3] at each of the four LV rings (A1=87 ms, A2=92 ms, A3=91 ms, A4=94 ms), we chose the A4 unipolar vector for LV pacing.

At the 1-month post-implant follow-up he showed worsening heart failure symptoms becoming NYHA class IV. Therefore, we performed an echocardiographic examination using contrast agent for the evaluation of intracavity blood motion at mechanical index of approximately 0.4 and high frame rates (70 to 90 Hz). The recorded clips of 1-month post-CRT implant, as well as of basal echocardiographic examination, were processed by echo-PIV, an optical method where the contrast agent bubbles are tracked from one frame to the next to calculate the instantaneous blood velocity fields. From these, the orientation angle (φ) of the global hemodynamic forces exchanged between blood and surrounding tissues is estimated. This angle ranges from zero, when flow force is predominantly along the base–apex direction, up to 90 degrees when it becomes transversal.

At pre-implant, the polar histogram showed the orientation and relative magnitude of blood-induced intraventricular forces were not properly aligned along the LV axis (highest φ; value 39.7°; Fig. 1: 3a); relative magnitude of blood-induced hemodynamic forces were not properly aligned along the LV axis (Fig. 1: 4a).

At 1-month post-CRT an echo-contrast examination was also performed during biventricular pacing protocol which provided the use of the four LV unipolar and A1–A4 pacing vectors. According to previous conjecture [1, 2], the biventricular pacing with the A1 unipolar LV vector (Fig. 1 Panel b; in the top right, arrow; see Additional files 2 and 3 for videos: acute study during biventricular pacing using the four LV unipolar and A1 vectors versus pacing off), showed the most longitudinal flow force (smallest φ; value 25.4°) and was then selected as optimal configuration for CRT pacing. At 6-months follow-up, 12-lead electrocardiogram (EKG) post-CRT demonstrated a QRS duration of 126 ms (Fig. 1: 1c), and the patient showed a significant improvement in NYHA class (II) and LV EF (53.2 %) with a significant reduction in ESV (57.1 ml versus 126 ml), and a low SDI (2.3 %) by RT3D-TTE approach (Fig. 1: 2c; see Additional file 4 for video); these settings provided a positive response to therapy, which was associated with improved alignment of intraventricular forces (Fig. 1: 3c); LV flow was more regular and the associated hemodynamic forces followed the base–apex orientation (Fig. 1: 4c).

## Discussion

Flow imaging, within the numerous limitations of the specific method employed here, may enable us to uncover a relationship between the quality of intraventricular vortex dynamics and cardiac function. LV flow represents an integral outcome of the tissue contraction/relaxation process whose dynamic features (local and short lasting) may not be easily detectable in terms of tissue displacement. Hemodynamic forces are known to participate in morphogenesis in embryonic hearts; therefore, they may also be one concurring factor during the pathological development of the grown heart [1]. The vortical hydrodynamic forces and their cytomechanical consequences by mechanosensing and mechanotransduction can radically affect ventricular remodeling with epigenetic nexus [4, 5]. The results of the only multi-center trial [6] in this field, led to the conclusion that no single echocardiographic measure of dyssynchrony may be recommended to improve patient selection for CRT due to the extreme variability of the collected data, and the applicability of dyssynchrony optimization in a “real-world” clinical setting is questionable. RT3D-TTE has shown an acceptable ability to assess left ventricular dyssynchrony, pre-CRT, with SDI [7], and to help guide lead placement that is concordant with the site of latest mechanical activation demonstrated with displays that are highly intuitive and desirable from an electrophysiologist’s perspective. However, high-quality images at high volume rate by RT3D-TTE are not always obtainable for patients with dilated cardiomyopathy, and the temporal resolution could hamper the analysis of small-scale variations of ventricular dyssynchrony and could thus influence the identification of appropriate pacing setting during acute echocardiographic optimization of left pacing vector. Moreover, current evidence does not strongly support the performance of atrioventricular (AV) and ventriculo-ventricular (VV) optimization routinely in all patients receiving CRT [8]. The analysis of flow dynamics inside the LV can provide new information about LV systolic and diastolic function through the analytical representation of the distribution of intraventricular pressure gradients; the assessment of morphological and energetic characteristics of fluid dynamics, both at baseline pre-implantation and after biventricular pacing, is potentially combinable with others parameters of echocardiographic methods that quantify LV systolic performance and residual systolic dyssynchrony respectively, to correct suboptimal device settings. In fact, it was previously suggested that fluid dynamics represents a sort of coupling between systole and diastole without a sharp separation between them [9]; this is because flow properties at one instant depend on the combination of mechanical events during previous time. The echo-PIV technique may be useful for elucidating the favorable effects of CRT on intraventricular fluid dynamics and it could be used to identify appropriate pacing setting during acute echocardiographic optimization of left pacing vector [2], with no relevant changes in electrical activation on EKG in the different LV pacing setting. This positive experience could be further tested in patients with narrow or intermediate QRS duration who may be expected to benefit from CRT, using echo-PIV analysis for measuring the extraordinary complexity of flow–wall relationship throughout the cardiac cycle.

## Conclusions

The observed changes in the orientation of flow momentum show an increasing longitudinal alignment in correspondence with an increasing volumetric response to CRT. This suggests that the electric changes provided by the therapy are more effective when they reflect into hemodynamic modifications that improve the longitudinal orientation of flow forces; conversely, a blood motion arrangement presenting transversal intraventricular forces is associated with lack of response to CRT. The echo-PIV technique may be useful for elucidating the favorable effects of CRT on intraventricular fluid dynamics and it could be used to identify appropriate pacing setting during acute echocardiographic optimization of quadripolar cardiac resynchronization device.

## Additional files

**Videos legend ECHO-PIV** “Two-dimensional TTE apical three chambers view with infusion of contrast agent was recorded for the evaluation of intracavity blood motion; LV flow vortex was assessed by ECHO-PIV bubbles tracking. The pressure gradient two-dimensional map shown with red pattern high pressure and with blue pattern low pressure respectively; the pressure gradient can be represented by flow momentum that is a quantity vector: product of velocity and mass. The polar graph explains pressure gradient distribution using quantitative parameter: angle between average direction of flow momentum and the long axis ventricle. This angle ranges from zero, when flow force is predominantly along the base–apex direction, up to 90 degrees when it becomes transversal. Changes in electrical activation setting modify the orientation of intraventricular forces during acute study”.

Additional file 1: RT3D-TTE full volume, one beat, pre-implant: very heterogeneous (orange regional pattern) dynamic map of the time minimum volume that looks at a 10 % of heart cycle time window when it moves through the heart cycle, with individual segments reaching end-systole at quite different times, and relative high Systolic Dyssynchrony Index (SDI) of 9.6 %. (WMV 161 kb)

Additional file 2: The intraventricular flow is dominated by longitudinal path of pressure gradient; the settings (A1-can) corresponding to the most aligned intraventricular forces are selected. (WMV 8.54 mb)

Additional file 3: The intraventricular flow is dominated by transversal path of pressure gradient and the relative magnitude of blood-induced hemodynamic forces are not properly aligned along the LV axis. (WMV 8.63 mb)

Additional file 4: RT3D-TTE full volume, one beat, post-CRT (6 m FU): low heterogeneous (orange regional pattern) dynamic map of the time minimum volume that looks at a 10 % of heart cycle time window when it moves through the heart cycle, with individual segments reaching end-systole at similar times, with low SDI of 2.3 %. (WMV 153 kb)
